# Non-use of dentures after tooth loss is associated with elevated sodium/potassium ratios in older adults: a retrospective cohort study

**DOI:** 10.3389/fdmed.2025.1479896

**Published:** 2025-04-24

**Authors:** Takafumi Abe, Tsuyoshi Hamano, Haruki Usuda, Koichiro Wada, Kenta Okuyama, Kazumichi Tominaga, Shozo Yano, Minoru Isomura

**Affiliations:** ^1^ Center for Community-Based Healthcare Research and Education (CoHRE), Head Office for Research and Academic Information, Shimane University, Shimane, Japan; ^2^ Department of Sports Sociology and Health Sciences, Faculty of Sociology, Kyoto Sangyo University, Kyoto, Japan; ^3^ Department of Pharmacology, Faculty of Medicine, Shimane University, Shimane, Japan; ^4^ Center for Primary Health Care Research, Lund University, Malmö, Sweden; ^5^ Tominaga Dental Office, Shimane, Japan; ^6^ Department of Laboratory Medicine, Faculty of Medicine, Shimane University, Shimane, Japan; ^7^ Faculty of Human Sciences, Shimane University, Shimane, Japan

**Keywords:** health checkups, tooth loss, Na/K ratio, older adults, salt intake

## Abstract

**Objective:**

An elevated sodium-to-potassium (Na/K) ratio in urine increases the risk of hypertension. Tooth loss in older adults can lead to a diminished nutritional state, including alterations in the Na/K ratio. However, the relationship between denture use and changes in the Na/K ratio among individuals with tooth loss has not been sufficiently explored. This study examined whether denture use modifies the association between tooth loss and changes in the Na/K ratio.

**Methods:**

Surveys in 2016 and 2018 included 473 older adults. The Na/K ratio was measured using spot urine tests. A dental hygienist evaluated the number of teeth and the use of dentures. We used generalized linear models to analyze the combined effect of the number of teeth and denture use on changes in the Na/K ratio.

**Results:**

Participants without dentures in the 0–19 teeth group showed a significant association with changes in the Na/K ratio (B = 0.635; 95% confidence interval = 0.038, 1.232) compared to those with ≥28 teeth, but denture users with 0–19 teeth did not show significant association. No significant association with changes in the Na/K ratio was observed in denture users and non-users with 20–27 teeth.

**Conclusion:**

In non-denture users with fewer than 20 teeth, the Na/K ratio was markedly elevated, but in denture users with fewer than 20 teeth it was not significant. These findings highlight the importance of promoting denture use in older adults with few remaining teeth to maintain their Na/K balance.

## 1 Introduction

According to Health Japan 21 (the second term) (1), the salt intake of the Japanese population is decreasing, but has not yet reached the target level. Additionally, vegetable intake has fallen short of the set goal, and improving the nutritional status of residents remains a public health priority in Japan. The 2017 American College of Cardiology/American Heart Association hypertension guidelines recommend increasing potassium and reducing sodium intake (2). The Japanese Society of Hypertension recently issued a consensus statement on the practical use and target value of the urine sodium-to-potassium (Na/K) ratio in assessing hypertension risk for Japanese individuals (3). Despite challenges in measurement accuracy (3–5), the measurement of the urine Na/K ratio, based on a combination of sodium and potassium levels in spot urine, is considered less invasive, resulting in minimal burden on participants. Additionally, spot urine test is a simpler test compared to the 24-h urine collection method (6). Consequently, the urine Na/K ratio is anticipated to be utilized as a novel indicator related to health (3, 7). In previous research, the Na/K ratio was found to be associated with blood pressure levels (8, 9), cardiovascular disease mortality, and all-cause mortality (10, 11). Therefore, reducing the dietary Na/K ratio is important to promote population health.

Oral conditions can impair dietary nutrient intake (12, 13). However, there is limited research examining the correlation between oral conditions and sodium and potassium levels. Marito et al. reported a negative association between the number of remaining teeth and masticatory performance and Na/K ratio (14). Nomura et al. reported no association between denture use and sodium or potassium (15). Both were cross-sectional studies; to the best of our knowledge, no longitudinal associations have been examined. Meanwhile, previous studies have shown that non-replaced missing teeth are associated with hypertension (16, 17). The association between poor oral conditions, such as the non-replaced missing teeth, and hypertension may potentially involve the Na/K ratio (14). In other words, the restoration of tooth loss with dentures could contribute to the enhancement of masticatory function and subsequent improvement in nutritional intake, potentially promoting an overall improvement in health status, including cognitive function (12, 18). Thus, we posited a hypothesis that the utilization of dentures would ameliorate the equilibrium between sodium and potassium concentrations in older adults who have experienced remarkable tooth loss. In this study, we examined whether denture use modified the association between tooth loss and changes in Na/K ratios in older Japanese adults.

## 2 Materials and methods

### 2.1 Study design and participants

This study was part of the Shimane Community-Based Healthcare Research and Education study. The Shimane Community-Based Healthcare Research and Education study is conducted to elucidate various factors contributing to lifestyle diseases in older adults (17, 19–21). This study employed data amassed during health checkups conducted in June 2016 and June 2018 in Okinoshima Town, Shimane, Japan. An aggregate of 842 older adults partook in the health examination in 2016 (baseline), and 502 participants were reevaluated in 2018 (follow-up). Upon the exclusion of nine participants due to incomplete information for study variables (Na/K ratio evaluated utilizing spot urine tests, *n* = 7; oral health status, *n* = 2), data from 473 participants were incorporated into the analysis. The study protocol received approval from the Medical Research Ethics Committee of Shimane University, Faculty of Medicine (approval number: 20051214-3), and adhered to the Code of Ethics of the World Medical Association (1964 Declaration of Helsinki and its subsequent amendments). Prior to enrollment in the study, written informed consent was procured from all participants.

### 2.2 Measurements

Sodium and potassium levels were assessed using spot urine tests. Spot urine was collected at the site of the health checkups (7, 19, 20). This test may have a lower precision compared to the 24 h urine collection method (3, 6); spot urine tests are practical in a large older population. Quantification of sodium and potassium concentrations was executed utilizing a BioMajesty 6070 G analyzer (JEOL Ltd., Tokyo, Japan). The Na/K ratio was computed by dividing the quantity of excreted sodium by the quantity of excreted potassium. Alterations in the Na/K ratio were determined by deducting the baseline data from the follow-up data (19).

This study used the number of teeth and denture use as indicators of oral health status. Intraoral examinations were performed by a trained dental hygienist, with the examiner and participants seated. The number of remaining teeth (functional teeth) was counted via oral visual examination. According to previous studies [9, 10], we divided the participants into three groups: individuals with ≥28, 20–27, and 0–19 teeth. Denture use (no or yes) was recorded by a dental hygienist.

In addition, a questionnaire was used to collect data on sex (male or female), age (years), smoking habits (no or yes), and alcohol consumption (no, sometimes, or daily; assessed from the question “How often do you drink?”) Body mass index was calculated from the recorded height and weight (kg/m^2^) and divided into three categories using Asian cut-off points (underweight: <18.5 kg/m^2^; normal: 18.5–22.9 kg/m^2^; overweight: ≥23.0 kg/m^2^) (22).

### 2.3 Statistical analysis

Regarding participant characteristics, frequency data are reported as numbers and percentages, and continuous data are presented as means and standard deviations. Generalized linear models (GLM) were used to estimate unstandardized regression coefficients (B) and 95% confidence intervals (CI) for changes in the Na/K ratio in relation to the combined variable of the number of teeth and denture use. To test the use of dentures as a potential effect modifier of the number of teeth and Na/K ratio, the number of teeth was entered together with denture use as a combined variable. The study participants were systematically classified into the following five groups, as reported by a previous study (17): (1) those possessing 28 or more teeth (serving as the reference group), (2) individuals with a tooth count ranging from 20–27 who utilize dentures, (3) participants with 20–27 teeth who do not use dentures, (4) participants with a tooth count between 0 and 19 who make use of dentures, and (5) those with 0–19 teeth who do not use dentures. We observed a moderate correlation between the number of teeth and denture use (*r* = 0.67; *P* < .001); therefore, considering multicollinearity, the number of teeth and denture use were included separately in the GLM. All analyses were adjusted for sex, age, body mass index, smoking habits, and alcohol consumption. All statistical analyses were performed using SPSS Statistics for Windows (version 29.0; IBM Corp., Armonk, NY). Statistical significance was set at *P* < .05.

## 3 Results

Table 1 presents participant characteristics. The number of participants with 0–19 teeth, 20–27 teeth, and 28 or more teeth (%) was 240 (50.7), 173 (36.6), and 60 (12.7), respectively. The mean (standard deviation) Na/K ratios at baseline, follow-up, and 2-year changes were 2.5 (1.4), 2.6 (1.5), and 0.1 (1.5), respectively.

**Table 1:** Participants' characteristics.

Variables	*n* or mean	(%) or SD
Sex		
Female	302	(63.8)
Male	171	(36.2)
Age		
≥80 years old	116	(24.5)
70–79 years old	211	(44.6)
≤69 years old	146	(30.9)
BMI		
Overweight ≥23.0 kg/m^2^	239	(50.5)
Normal 18.5–22.9 kg/m^2^	210	(44.4)
Underweight <18.5 kg/m^2^	24	(5.1)
Smoking habits		
Yes	21	(4.4)
No	452	(95.6)
Alcohol consumption		
Every day	109	(23.0)
Sometimes	49	(10.4)
No	315	(66.6)
Number of teeth		
≥28 teeth	60	(12.7)
20–27 teeth	173	(36.6)
0–19 teeth	240	(50.7)
Denture use		
Yes	256	(54.1)
No	217	(45.9)
Combination of number of teeth and denture use		
≥28 teeth	60	(12.7)
20–27 teeth with use of dentures	45	(9.5)
20–27 teeth without use of dentures	128	(27.1)
0–19 teeth with use of dentures	210	(44.4)
0–19 teeth without use of dentures	30	(6.3)
Urinary Na/K ratio		
At baseline	2.5	1.4
At follow-up	2.6	1.5
2-Year Changes^a^	0.1	1.5

SD, standard deviation; BMI, body mass index; Na/K, sodium-to-potassium.

^a^ Changes in the Na/K ratio were calculated by subtracting the baseline data from the follow-up data.

**Figure 1:**
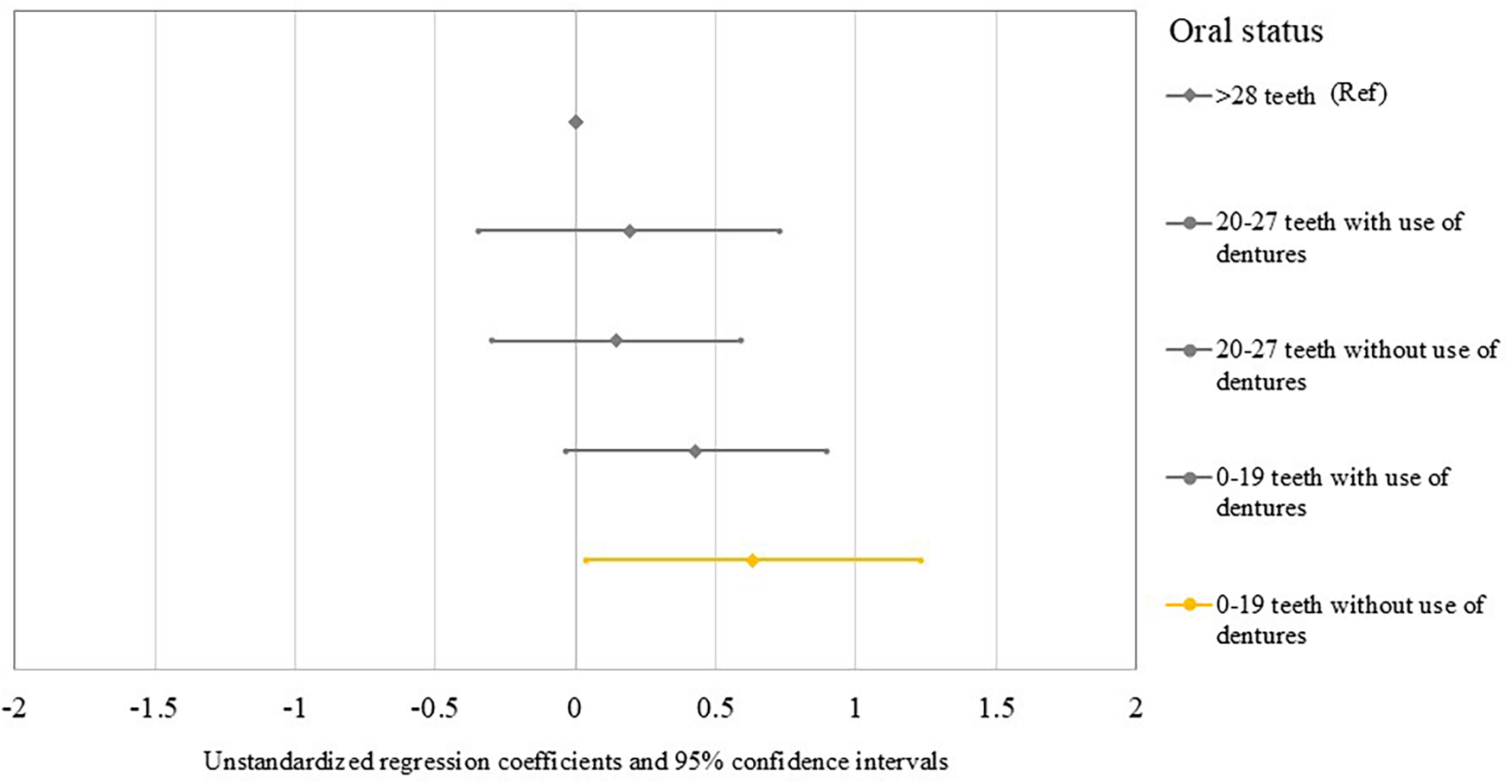
Forest plots show the unstandardized regression coefficients and 95% confidence intervals for changes in the Na/K ratio in relation to the combined variable of the number of teeth and use of dentures.

Table 2 and Figure 1 show the association between the combined variable of number of teeth and denture use and changes in the Na/K ratio. When using ≥28 teeth as the reference group, a significant increase in the Na/K ratio was observed in participants without dentures with 0–19 teeth (B = 0.635; 95% CI = 0.038, 1.232; *P* = .037), but denture users with 0–19 teeth did not show significant association with changes in the Na/K ratio (B = 0.431; 95% CI = −0.034, 0.896; *P* = .069). No significant association with changes in the Na/K ratio was observed in denture users (B = 0.192; 95% CI = −0.344, 0.728; *P* = .483) or non-users (B = 0.145; 95% CI = −0.300, 0.589; *P* = .523) with 20–27 teeth.

**Table 2:** Association between combined variable of number of teeth and use of dentures and changes in sodium-to-potassium ratio among older adults.

Number of teeth	Denture use	B	95% CI	*P* value
≥28 teeth		ref		
20–27 teeth	With use of dentures	0.192	(−0.344, 0.728)	0.483
	Without use of dentures	0.145	(−0.300, 0.589)	0.523
0–19 teeth	With use of dentures	0.431	(−0.034, 0.896)	0.069
	Without use of dentures	0.635	(0.038, 1.232)	0.037

B, unstandardized regression coefficients; CI, confidence interval.

Generalized linear model was adjusted for sex, age, body mass index, smoking habits, and alcohol consumption. Statistical significance was set at *P* < .05.

## 4 Discussion

This study examined whether the possible modification of the association between tooth loss and changes in the Na/K ratio in older adults was due to the use of dentures. When there are few remaining teeth (0–19 teeth), the Na/K ratio may increase when dentures are not used. However, in denture users with fewer than 20 teeth, this increase was not significant. These results suggest that conducting educational training for oral health may impact the nutritional status through regular denture maintenance (23). In addition, dentures can help restore esthetics, enhancing social interactions in older adults (24). However, there may be instances where the use of dentures is avoided owing to poor quality and discomfort, such as pain (25). In particular, occluding pairs may contribute to masticatory function more than the number of remaining teeth (26). Therefore, from the perspectives of sociability and functionality, it is necessary to regularly review the condition of the dentures.

A previous study by Marito et al. reported negative associations between oral health and the Na/K ratio in 894 older Japanese adults (14). In their study, calibrated dental examiners assessed the number of remaining teeth in each participant, and Na/K ratios were estimated using a brief self-administered diet history questionnaire. Their bivariate correlation analysis resulted in a correlation coefficient of −0.07 (*P* = .03). Although our study employed different measurement and analysis methods, it supports the concept that tooth scarcity increases the Na/K ratio. Nomura et al. conducted a study involving 701 older Japanese individuals examining denture usage through questionnaires and sodium or potassium intake using the brief self-administered diet history questionnaire (15). The results showed no difference in sodium or potassium intake based on denture use. Therefore, although previous studies have not created combined variables for tooth loss and denture use—making comparisons difficult—our findings provide a new perspective on the relationship between nutritional status, the number of teeth, and the use of dentures.

Potential mechanisms that could elucidate the association between tooth loss and alterations in the Na/K ratio have been put forth. The masticatory function in older adults possessing fewer than 20 residual teeth is reportedly inferior compared to their counterparts with a greater number of teeth. However, the utilization of denture prostheses could potentially aid in the restoration of masticatory function (27). The utilization of dentures may be linked to variations in the nutritional status of older adults experiencing extensive tooth loss (28). Those with significant tooth loss in later life commonly encounter challenges in consuming raw fruits and vegetables, given the increased masticatory effort required compared to other food types (29, 30). This may limit their overall food choices, eventually leading to health problems such as hypertension through nutritional imbalances (28, 29). Lee et al. reported that poor nutritional condition, attributed to challenges in food selection and consumption, has been identified as a mediating factor in the relationship between unsatisfactory masticatory function and an elevated risk of mortality (31). Furthermore, mastication with dentures may help maintain hippocampal activity, potentially playing a role in preserving cognitive abilities and memory (32). Additionally, the presence and functioning of periodontal mechanoreceptors in the teeth during mastication are crucial (33). The removal of these receptors during tooth extraction leads to a decline in masticatory ability. Maintaining periodontal mechanoreceptor activity, which depends on the type of implants or dentures used in treatment, is also an important aspect for ensuring proper nutritional intake. Therefore, considering both oral conditions and dietary Na/K ratios is important to promote population health, especially among older adults.

### 4.1 Limitations

First, we could not establish a strong causal relationship because this study employed a retrospective design. Second, this study was exclusively composed of inhabitants from specific areas who engaged in health checkups. Furthermore, Okinoshima, the town under study, is an island with a higher likelihood of food choices being limited. This may be particularly significant in cases where oral function is diminished. Although the findings of this study may be challenging to generalize because of a potential sampling bias, these results may represent a significant step toward understanding potential issues related to regional disparities and limited food environments. Third, the results drawn in our study are reported on a limited sample size; therefore, the statistical power was low. Fourth, the spot urine test used to measure sodium and potassium levels in the present study may have a lower precision compared to the 24 h urine collection method (6). However, when applying the 24 h urine collection method to older adults, there is a potential for collection errors. Spot urine testing offers the advantage of reducing the burden on older adults. Moreover, in order to investigate the dietary content of the participants, it is necessary to utilize Dietary Records (34), 24 h Dietary Recalls (35), and the Food Frequency Questionnaire (36) as dietary surveys other than urine tests. By doing so, there is a possibility that future research can clarify the association between tooth loss and changes in nutrition based on specific dietary content. Fifth, we did not investigate whether the dentures were partial or complete. Therefore, it cannot be ruled out that this difference may have influenced the results (33). Finally, the unmeasured variables were not included as confounders. For example, a study in the United States has reported a correlation between potassium levels and diet costs (37). This suggests that the economic status of older adults may influence food choices. Therefore, future studies should examine the influence of additional variables [e.g., educational level (38) and social isolation (39)]. Based on the above considerations, future research should include large-scale observational studies as well as randomized controlled trials to investigate whether the use of dentures (treatment) can improve dietary habits (40), such as increasing vegetable intake, and thereby maintaining a favorable Na/K ratio.

### 4.2 Conclusion

This study showed that the non-use of dentures appeared to worsen the adverse effects of tooth loss on the Na/K ratio, whereas the Na/K ratio did not increase in denture users with tooth loss. These findings highlight the importance of promoting denture use in older adults with few remaining teeth to maintain their Na/K balance. Future research is needed to investigate whether the use of dentures, that is, oral care, can improve dietary habits and maintain a favorable Na/K ratio.

## Data Availability

The datasets presented in this article are not readily available because The consent of the participants did not include a provision for the data to be shared publicly. Requests to access the datasets should be directed to Medical Research Ethics Committee, Shimane University Faculty of Medicine (kenkyu@med.shimane-u.ac.jp).
